# Impact of Statewide Program To Promote Appropriate Antimicrobial Drug Use

**DOI:** 10.3201/eid1106.050118

**Published:** 2005-06

**Authors:** Edward A. Belongia, Mary Jo Knobloch, Burney A. Kieke, Jeffrey P. Davis, Carolyn Janette, Richard E. Besser

**Affiliations:** * Marshfield Clinic Research Foundation, Marshfield, Wisconsin, USA;; † Wisconsin Division of Public Health, Madison, Wisconsin, USA;; ‡ Wisconsin Medical Society, Madison, Wisconsin, USA;; § Centers for Disease Control and Prevention, Atlanta, Georgia, USA

**Keywords:** drug resistance, drug utilization, drug trends, health education, outcome and process assessment, respiratory tract infections, drug therapy, physician

## Abstract

The Wisconsin Antibiotic Resistance Network (WARN) was launched in 1999 to educate physicians and the public about judicious antimicrobial drug use. Public education included radio and television advertisements, posters, pamphlets, and presentations at childcare centers. Physician education included mailings, susceptibility reports, practice guidelines, satellite conferences, and presentations. We analyzed antimicrobial prescribing data for primary care physicians in Wisconsin and Minnesota (control state). Antimicrobial prescribing declined 19.8% in Minnesota and 20.4% in Wisconsin from 1998 to 2003. Prescribing by internists declined significantly more in Wisconsin than Minnesota, but the opposite was true for pediatricians. We conclude that the secular trend of declining antimicrobial drug use continued through 2003, but a large-scale educational program did not generate greater reductions in Wisconsin despite improved knowledge. State and local organizations should consider a balanced approach that includes limited statewide educational activities with increasing emphasis on local, provider-level interventions and policy development to promote careful antimicrobial drug use.

Antimicrobial drug–resistant strains of community-acquired pathogens, including *Streptococcus pneumoniae* and *Staphylococcus aureus*, have emerged as serious global health threats (1–3). Multiple studies have demonstrated a strong and consistent link between antimicrobial drug use and antimicrobial resistance at both individual and population levels (4–9). Despite this link, inappropriate and ineffective use of antimicrobial agents for viral respiratory infections is common (10–16). In 1998, the Institute of Medicine issued a workshop report that addressed the growing problem of antimicrobial drug resistance and potential strategies to prolong the effectiveness of existing drugs (17). The report found that physicians and patients have not received adequate information about the appropriate use of antimicrobial drugs and the short- and long-term risks of overuse. Several approaches were suggested, including multifaceted clinician education, clinical practice protocols, feedback on local resistance trends, patient-oriented educational materials, and use of popular media for public education.

We describe the activities and impact of a 5-year, multifaceted educational campaign to reduce outpatient antimicrobial drug prescribing in Wisconsin. The Wisconsin Antibiotic Resistance Network (WARN) was launched in 1999 as a federally funded demonstration project to educate primary care physicians and the public about drug resistance and judicious antimicrobial drug use. Antimicrobial drug prescribing rates were assessed annually and compared to Minnesota prescribing rates, where educational activities were limited before 2002.

## Methods

### WARN Organization

WARN was established as a collaborative project involving the Marshfield Clinic Research Foundation, Wisconsin Medical Society, and Wisconsin Division of Public Health. The Wisconsin Medical Society collaborated with the Marshfield Clinic Research Foundation to develop the public education campaign, and the latter organization was responsible for data collection and evaluation. The Division of Public Health assisted with program planning and collected invasive pneumococcal isolates for susceptibility testing.

For most of the project period, WARN employed 2 program managers and 2 health educators. Representatives of each position were based in Madison and in Marshfield to cover the southern and northern parts of the state. An advisory board was established with representation from managed care organizations, employers, local public health agencies, primary care practices, infectious disease experts, childcare centers, pharmacists, and the Centers for Disease Control and Prevention. Campaign themes, characters, messages, and print materials were developed during the first year (1999). An expert panel was convened to develop local clinical practice guidelines for acute respiratory illness in Wisconsin. Educational needs were assessed through surveys of primary care clinicians and the general public (18). Limited public and physician educational activities were initiated in 2000, and the program was implemented in full from 2001 to 2003. Educational interventions were designed to be multifaceted and consistent with behavioral research that suggested a need for a variety of educational strategies with repetitive and reinforcing messages (19–21).

### Public and Physician Education

The tag lines for the public education campaign were "There's no excuse for overuse!" and "Get smart about antibiotics!" The campaign mascots, Annie Biotic and Moxie Cillin, were cartoon characters designed to appeal largely to children and parents. Public education materials included posters, brochures, stickers, coloring sheets, magnets, and disease-specific parent handouts (Table 1). All materials were available free of charge to healthcare providers, clinics, and community organizations. Spanish translations were also available for many of the written materials. A Web site was established through the Wisconsin Medical Society to describe WARN activities and facilitate ordering materials. The distribution volume for WARN educational materials is shown in Table 2.

**Table 1:** Major WARN activities and initiatives, 2000–2003*

Activity	2000	2001	2002	2003
Public education				
Mailing to all licensed family and group childcare providers		X	X	X
Exhibits at health fairs and community events	X	X	X	X
Public appearances by costumed characters	X	X	X	X
Distribution of sample materials to pharmacies	X			
Slide presentations at childcare centers	X	X	X	X
Newsletter copy distributed to healthcare organizations	X	X		
WARN paycheck stuffers for state employees	X		X	X
Slide presentations for community and state organizations	X	X	X	X
Physician education				
Sample materials and order form mailed to >8,000 licensed primary care clinicians and pharmacists		X	X	X
Satellite broadcasts on management of respiratory illness	X	X		
Academic detailing packets distributed to health plans, clinics, public health staff	X	X	X	X
Narrated slide presentation on CD-ROM mailed to primary care clinicians		X		
Presentations at healthcare facilities, professional meetings, and conferences	X	X	X	X
Distributed invasive pneumococcal susceptibility report for Wisconsin	X	X	X	X
Distributed original (2000) and revised (2002) clinical practice fact sheets to ≈9,000 clinicians and pharmacists	X		X	
WARN resource binder distributed to 16 health plans		X		
Media campaign				
Advertisements on Wisconsin Radio Network or Wisconsin Public Radio	X	X	X	X
Guest editorials in newspapers	X	X	X	X
Governor declared "Get Smart About Antibiotics Month"	X			
Television advertisements—Dick Van Dyke and Bill Nye			X	X
Earned media—radio and/or television news coverage	X	X	X	X
Earned media—newspaper coverage	X	X	X	X

*WARN, Wisconsin Antibiotic Resistance Network.

**Table 2:** Distribution of WARN educational materials (2000–2003)*

Type of material	Approximate no. distributed
WARN parent brochures	700,000
WARN posters	26,000
CDC posters (new in 2003)	900
CDC adult brochures	400,000
Spanish-language posters	5,000
Viral illness card	18,000
CDC viral prescription pad (new in 2003)	300
Parent illness handouts	23,000
Coloring sheets	450,000
Stickers	620,000
Magnets	50,000
Clinical practice fact sheets for respiratory illness	20,000
Pneumococcal susceptibility reports	38,000

*WARN, Wisconsin Antibiotic Resistance Network; CDC, Centers for Disease Control and Prevention.

Outreach to childcare centers was a major focus of community education because children attending group child care have high rates of respiratory illness. Annual mailings were sent to >5,000 licensed childcare centers, and on-site presentations were given at 170 centers. Additional presentations were made at childcare conferences and in college classes on early childhood education. Physician-education activities began in 2000 and included direct mailings with samples of WARN materials, development and distribution of guidelines for judicious antibiotic use, satellite broadcasts, dissemination of pneumococcal antimicrobial resistance data, and professional presentations (Table 1).

### Media Campaign

The media campaign included paid advertising and strategies to maximize coverage. The campaign was launched with a press conference in 1999, followed by repeated news media coverage of WARN and antimicrobial resistance issues from 2000 through 2003 (Table 1). This coverage included newspaper reports in Milwaukee, Madison, and multiple smaller communities, as well as local television news stories in Milwaukee, Madison, and Wausau. Paid advertising was initiated on radio stations broadcasting throughout the state and on television stations. Because of the high cost, television advertisements were limited to periods of 2 to 4 weeks during the peak respiratory illness season.

### Research Design and Outcome Measures

Multiple evaluation components were developed to measure the effect of the WARN educational campaign. The major outcome for this report was annual antimicrobial drug use, measured by the number of primary care prescriptions and the volume of retail antimicrobial drug sales.

During the late 1990s, a national secular trend of declining antimicrobial drug use occurred (22–24). To distinguish the impact of WARN from the secular trend, antimicrobial drug–prescribing measures were obtained for both Wisconsin and Minnesota. Although Minnesota is not representative of the entire country, the use of a comparison state provided the opportunity to distinguish intervention-related changes from regional trends in use that were unrelated to the intervention. Minnesota was chosen for geographic proximity and similarity in terms of population size and ethnic distribution. Before 2002, patient educational activities in Minnesota were limited. A group of 6 Minnesota managed care plans distributed ≈17,000 cough and cold kits to patients during the 2000–2001 respiratory illness season, and 31,000 kits during the 2001–2002 season. An article promoting appropriate antimicrobial drug use was published in the April 2001 issue of Minnesota Medicine (25), but no other formal programs to educate Minnesota physicians on appropriate antimicrobial drug use were implemented until late 2002, when sample materials (posters, buttons, "prescription pads" for symptomatic therapy) were mailed to managers at 377 Minnesota clinics and urgent care centers.

### Measures of Antimicrobial Drug Use

Prescribing data and retail volume distribution data (measuring retail sales) were obtained from a commercial source (IMS Health, Inc., Plymouth Meeting, PA, USA) for the states of Wisconsin and Minnesota. Physician prescribing data were available for 1998 and 2000 through 2003. Prescribing and volume distribution data were not available for individual drugs within each class. The source data did not include any information regarding the specific diagnosis or patient characteristics.

The prescribing databases included only new outpatient prescriptions, and they were derived from transactional data provided by 59% of all retail pharmacies in Wisconsin and Minnesota. Approximately 65% of chain pharmacies and 51% of independent retail pharmacies contributed raw prescribing data. Prescriptions from unsampled stores were estimated on the basis of prescription totals from matched nearby stores, with weighting to adjust for differences in total retail sales volume, which was available for nearly all stores. Estimates were also weighted to account for the distance between sampled stores and matched unsampled stores, with closer stores contributing more to the estimated prescription volume. The proportion of all prescriptions in each state that were based on estimated data from unsampled stores was 33%–37%.

Physician-level prescribing data included data for all licensed physicians with any of the following primary specialty codes: family (and general) practice, internal medicine, pediatrics, or emergency medicine. Physicians were classified geographically as practicing within or outside of the largest metropolitan statistical area in each state. These 2 metropolitan statistical areas were Milwaukee-Waukesha (4 counties) and Minneapolis-St. Paul (11 counties).

Retail volume distribution was determined by the volume of antimicrobial drugs distributed to retail outlets on a monthly basis from 1999 to 2002. This distribution was derived from an independent data source relative to the physician-level prescribing data. Retail distribution data (measured in kilograms) were reported by wholesalers and distributors serving pharmacies in both states. Retail volume was not linked to specific prescriptions or providers and therefore represented a measure of total outpatient antimicrobial drug use in each state. Volume was based on distribution to retailers rather than actual sales to patients, and distributed drugs could be returned to wholesalers without being sold. In this situation, returned drugs were subtracted from the total distributed in a given month to yield the net retail distribution for each drug class. Inpatient pharmacies, prisons, veterinary offices, nursing homes, dialysis clinics, and federal government sites were excluded from the volume distribution data. The retail volume sales database captured 93% of actual antimicrobial drug distribution in Wisconsin and Minnesota. Volume sales were divided by the annual population estimates in each state and reported as grams per capita.

The following product groups were included in the assessment of outpatient prescribing and retail volume sales: amoxicillin/penicillin, amoxicillin-clavulanate, cephalosporins, macrolides, extended-spectrum macrolides (azithromycin, clarithromycin), fluoroquinolones, tetracyclines, trimethoprim-sulfamethoxazole, and other sulfa drugs (including erythromycin/sulfisoxazole). Solid and liquid formulations were reported separately, and liquid formulations were used as surrogates for pediatric prescribing. Broad-spectrum antimicrobial drugs were also analyzed separately. Although no standard definition of broad spectrum antimicrobial drugs exists, a previous report on national trends in antimicrobial drug use classified the following groups as broad-spectrum: extended-spectrum macrolides, fluoroquinolones, second- and third-generation cephalosporins, and amoxicillin-clavulanate (23). We used the same classification with 1 exception. In this study we classified all cephalosporins as broad-spectrum because we were unable to distinguish first-, second-, and third-generation cephalosporins.

### Prescriber Cohort

A cohort of primary care physicians was established to monitor and compare longitudinal trends in prescribing antimicrobial drugs. The cohort was defined as primary care physicians in Minnesota and Wisconsin who prescribed at least 1 antimicrobial drug in each month during the baseline year (1998) and each of the follow-up years (2000–2003). This criterion was used to avoid including residents and other physicians in temporary practice settings. It also avoided including nonpractice time in prescribing rate calculations, since information on individual practice patterns was not available. The annual antimicrobial prescribing rate was calculated by dividing the number of new, filled prescriptions by the number of physicians in the cohort.

The original 1998 prescriber database included 9,164 primary care physicians in Minnesota or Wisconsin who prescribed any antimicrobial drug. Of those, 4,115 (45%) prescribed ≥1 antimicrobial drug per month in 1998 and annually from 2000 through 2003. This group made up the final cohort for analysis of longitudinal prescribing trends. A secondary analysis of prescribing trends was performed based on the larger group of primary care physicians (n = 12,790) who prescribed ≥1 antimicrobial drugs during any of the follow-up years but who were not necessarily in continuous practice.

## Statistical Analysis

Prescribing rates for the 4,115 physicians in practice throughout the study period were computed as the number of antimicrobial prescriptions in a given year divided by the number of physicians. Prescribing rates were therefore equivalent to the mean number of prescriptions for antimicrobial drugs per physician each year. Population-based or patient-based prescribing rates could not be calculated because the populations served by the prescribing cohort were undefined. To compare prescribing rates for each year of the intervention period (2000–2003) to baseline (1998), we fit Poisson regression models. Generalized estimating equations (GEE) were employed to account for within-physician correlation (26). An autoregressive working correlation structure was specified in the Poisson-GEE models, and appropriate steps were taken to accommodate the absence of data for calendar year 1999. Statistical comparisons of changes in rates in Wisconsin versus those in Minnesota were derived from these models.

Differences in baseline antimicrobial prescribing were addressed by fitting additional Poisson-GEE models using data from the intervention period with the natural log of the baseline number of antimicrobial drug prescriptions included as an independent variable. Prescribing rates per physician-month were computed when analyzing the 12,790 physicians who wrote such prescriptions in ≥1 month of the study period. Months where a physician wrote at least 1 prescription for an antimicrobial drug were included in the denominators of rates (i.e., a physician was assumed not to be in practice in months when he did not write any such prescriptions). Per capita antimicrobial drug sales were calculated based on annual population estimates (www.census.gov). Analyses were completed by using the SAS software (SAS Institute Inc., Cary, NC, USA).

## Results

**Figure 1:**
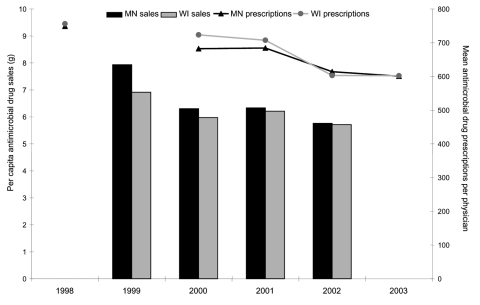
Temporal trends in per capita antimicrobial drug sales and the mean number of prescriptions per physician in Minnesota and Wisconsin.

The 4,115 primary care physicians in long-term practice included 2,009 (49%) in Minnesota and 2,106 (51%) in Wisconsin. The proportion of physicians in family practice was higher in Wisconsin, and the proportion in internal medicine was higher in Minnesota (Table 3). During 1998, these physicians generated 1.5 million prescriptions for antimicrobial drugs in each state, and the crude antimicrobial prescribing rate was nearly identical across states. From 2000 through 2003, the prescribing rate for antimicrobial drugs in both states gradually declined (Figure 1). From 1998 to 2003, the antimicrobial prescribing rate was reduced by 19.8% in Minnesota and by 20.4% in Wisconsin. The percentage reduction was 19.5% and 18.6%, respectively, in the secondary analysis, which included the 12,790 primary care physicians who prescribed antimicrobial drugs at any time during the follow-up period.

**Table 3:** Characteristics of 4,115 primary care physicians in long-term practice, Minnesota and Wisconsin

Characteristic*	Minnesota, n (%)	Wisconsin, n (%)
Specialty		
Family practice†	1,245 (62.0)	1,043 (49.5)
Internal medicine	396 (19.7)	565 (26.8)
Pediatrics	256 (12.7)	309 (14.7)
Emergency medicine	112 (5.6)	189 (9.0)
Practice location‡		
Milwaukee-Waukesha MSA	–	726 (34.8)
Minneapolis-St. Paul MSA	1,216 (61.5)	–
Other counties	762 (38.5)	1,363 (65.2)

*MSA, metropolitan statistical area. †Includes 67 physicians in general practice. ‡48 physicians excluded because practice location category changed during follow-up period.

Retail sales of antimicrobial drugs (grams per capita) declined by 27.4% in Minnesota and 17.3% in Wisconsin from 1999 through 2002 (Figure 1). Sales of amoxicillin and penicillin exceeded those of other product groups in each year and accounted for 37% of all retail antimicrobial distribution. In both states, retail sales of fluoroquinolones remained level, and sales of amoxicillin-clavulanate increased. Sales of most other product groups declined by 15% or more.

Stratification of antimicrobial prescribing rates by specialty showed that the reduction in prescribing differed only for physicians in internal medicine and pediatrics (Table 4). Antimicrobial prescribing declined more in Wisconsin than Minnesota among internal medicine physicians, but the opposite was true for pediatricians. In Minnesota, the reduction in antimicrobial use was similar for physicians practicing in the Minneapolis–St. Paul metropolitan statistical area and those practicing elsewhere in the state (Table 5). In Wisconsin, the reduction in antimicrobial prescribing was much less in the Milwaukee-Waukesha metropolitan statistical area than in the remainder of the state. Antimicrobial prescribing declined significantly more in the Minneapolis-St. Paul metropolitan statistical area than in the corresponding Milwaukee-Waukesha area .

**Table 4:** Changes in antimicrobial drug prescribing by specialty and state, 1998–2003*

Specialty	Wisconsin prescribing rate			Minnesota prescribing rate			
	1998	2003	% reduction	1998	2003	% reduction	p value†
Family practice	810	631	22	843	685	19	0.11
Internal medicine	540	447	17	366	329	10	0.03
Pediatrics	1,126	891	21	1,068	751	30	0.006
Emergency medicine	519	451	13	306	303	1	0.25

*The annual antimicrobial drug prescribing rate was calculated by dividing the number of new filled prescriptions by the number of prescribers in each specialty. †p value for comparison of reduction in Wisconsin vs. Minnesota.

**Table 5:** Changes in prescribing rate for antimicrobial drugs, by practice location, 1998–2003*

Practice location	Wisconsin prescribing rate			Minnesota prescribing rate			
	1998	2003	% reduction	1998	2003	% reduction	p value†
Major metropolitan area‡	719	639	11	711	568	20	<0.001
Other areas of Minnesota and Wisconsin	778	583	25	814	657	19	0.005

*The annual prescribing rate for antimicrobial drugs was calculated by dividing the number of new filled prescriptions by the number of prescribers in each specialty. †p value for comparison of reduction in Wisconsin vs. Minnesota. ‡Includes the Milwaukee-Waukesha metropolitan statistical area (4 counties) for Wisconsin and the Minneapolis-St. Paul metropolitan statistical area (11 counties) for Minnesota. Two Wisconsin counties were excluded from the latter area.

Prescriptions for liquid antimicrobial drugs (a surrogate for pediatric use) declined 29% in Wisconsin and 32% in Minnesota from 1998 to 2003 (p = 0.17). Prescriptions for solid formulations declined 15%–17% in each state. The percentage of prescriptions for broad-spectrum agents was unchanged (within 2%) in each state from 1998 through 2003.

**Figure 2:**
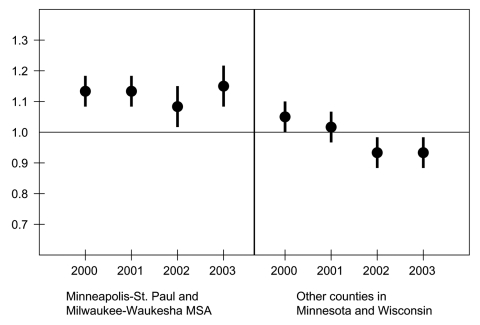
Antimicrobial prescribing rate ratios by year and practice location, adjusted for specialty and baseline (1998) prescribing rate. The vertical bars show 95% confidence intervals. Ratios <1 indicate lower antimicrobial prescribing by Wisconsin physicians relative to Minnesota physicians. MSA, metropolitan statistical area.

Regression models were fit to compare prescribing rates in Wisconsin and Minnesota during each year from 2000 to 2003, with adjustment for specialty and baseline prescribing in 1998. Two separate models were generated. The first included physicians practicing in the Minneapolis–St. Paul metropolitan statistical area or the Milwaukee-Waukesha metropolitan statistical area. The second included physicians practicing in other counties of Minnesota or Wisconsin. In the latter model, the prescribing rate ratio was <1 in 2002 and 2003, indicating that, when the 2 largest metropolitan areas were excluded, Wisconsin physicians had significantly lower prescribing rates than those in Minnesota (Figure 2). In contrast, the prescribing rate ratio was >1 in each year within the 2 largest metropolitan areas, indicating that physicians in the Milwaukee-Waukesha MSA had higher prescribing rates than those in the Minneapolis-St. Paul MSA, after adjusting for specialty and baseline prescribing.

## Discussion

WARN represents the largest program on appropriate antimicrobial drug use that has been evaluated to measure the effect on prescribing. Previously published studies demonstrated that interventions at the level of the physician, clinic, or community had a modest effect on prescribing (20,27–32). These focused programs are useful in evaluating specific intervention strategies in a relatively controlled setting. However, adoption of new practices may be slow even when the intervention is proven to be effective, and generalizability to larger populations may be limited. In contrast, large-scale programs can reach physicians and the general public in an entire state or large metropolitan area. The development of WARN reflected a need to implement and evaluate a large-scale demonstration project that could influence antimicrobial prescribing throughout Wisconsin in a relatively short time. At the time WARN was conceived and funded, knowledge of national trends in antimicrobial prescribing was limited, but we now know that prescribing declined substantially for both pediatric and adult populations in the 1990s (22–24,33).

Outpatient antimicrobial use declined substantially in both Wisconsin and Minnesota from 1998 to 2003, and no additional intervention-related effect was apparent in Wisconsin. Secondary analyses by specialty and practice location demonstrated variable reductions in prescribing of antimicrobial drugs, but to what extent these reductions were related to WARN interventions, as opposed to other factors that may be influencing secular trends, is unclear. The potential effect of these other factors is illustrated by the observation that Minnesota pediatricians improved their prescribing practices more than Wisconsin pediatricians, despite the absence of an organized, large-scale program to improve pediatric prescribing in Minnesota.

We found that changes in antimicrobial drug use were less pronounced in the Milwaukee-Waukesha metropolitan area than in other regions of Wisconsin. This finding contrasts with findings in Minnesota, where the decrease in antimicrobial drug use in the Minneapolis-St. Paul metropolitan area was similar to that in the rest of the state. When these large metropolitan areas were excluded from the analysis, prescribing of antimicrobial drugs decreased more in Wisconsin than in Minnesota. Several factors may have contributed to the relatively low impact of WARN in the Milwaukee area. These factors include the absence of paid staff working in Milwaukee, few connections with the Milwaukee medical community, and the large number of clinical practices and health plans.

Funding for WARN exceeded levels for other state-based programs on antimicrobial drug resistance, but it was far lower than the funding level for some other public education campaigns. Annual funding for WARN staffing, programs, and materials (excluding indirect costs and evaluation activities) was ≈$238,000–$342,000. By comparison, Wisconsin receives ≈$10 million in state funding and $1.1 million in federal funding each year for smoking prevention programs (Maureen Busalacchi, pers. comm.). WARN funding for education on antimicrobial resistance was not sufficient to conduct a widespread and sustained media campaign, although whether such a campaign would have led to further reductions in antimicrobial use is not known.

One other published study reported the effect of interventions promoting appropriate antimicrobial drug use in a large, highly populated area. In Knox County, Tennessee, a 12-month multifaceted campaign was conducted in 1997 and early 1998 (34). The clinician intervention included professional presentations, distribution of pediatric principles of judicious antimicrobial drug use (35), and newsletter articles. Patient and public education included distribution of print materials, news media coverage, and public service announcements. In the Medicaid managed care program, antimicrobial drug prescribing for respiratory illness declined 19% in Knox County and 8% in the control counties compared to the previous year (intervention attributable effect of 11%, p<0.001). The Knox County intervention was smaller in scale and shorter in duration than WARN, and the generalizability to non-Medicaid populations is unknown.

Evaluating large-scale, multifaceted educational programs such as WARN has several limitations. The control population in Minnesota was not isolated, and educational materials and messages may have diffused into the control area from a variety of sources. For example, during the WARN follow-up period, national guidelines on appropriate antimicrobial use were published and endorsed by the Centers for Disease Control and Prevention and major professional organizations (35–37). WARN also reached limited populations in Minnesota: radio advertisements included areas of eastern Minnesota, and some Minnesota physicians received the WARN satellite broadcasts on appropriate antimicrobial drug use. In addition, efforts within Minnesota to improve prescribing of antimicrobial drugs increased during the follow-up period, particularly during 2002 and 2003. A single state may not be the optimal comparison population, and Minnesota in particular has a highly educated population and a proactive public health infrastructure with strong ties to the healthcare delivery system. As a result, the improvements in antimicrobial drug prescribing within Minnesota may have been greater than those in many other states. Finally, the commercial prescribing data used for the WARN evaluation did not include any information on visits or diagnoses. We therefore cannot determine if the declines in antimicrobial prescribing in Wisconsin and Minnesota were associated with a declining rate of visits for acute respiratory illness or if the rate of prescribing declined for specific diagnoses.

The centralized development of WARN programs and materials facilitated statewide distribution, but it also limited the level of clinician involvement at the local level. Direct, face-to-face communication with physicians was rarely possible. In contrast, practice-level interventions have shown modest success, and we speculate that these focused, participatory interventions may promote physician behavior change more directly than a mass education campaign such as WARN. However, WARN succeeded in changing physicians' knowledge and attitudes regarding appropriate antimicrobial drug use, and WARN materials were widely used by primary care clinicians throughout the state. In a survey of primary care clinicians, 90% of respondents had heard of WARN, and 70% of those had used WARN materials for patient education (38). Models of behavior change suggest that changes in prescribing behavior are preceded by important cognitive changes that proceed in stepwise fashion (39). Improvements in knowledge and beliefs among both physicians and patients may therefore be markers of progress, which may facilitate the future success of provider-level interventions developed by clinics and managed care organizations in Wisconsin.

Increased funding for state-level educational campaigns to promote appropriate antimicrobial drug use does not appear warranted by the results of this evaluation. However, the combined effect of national guidelines for appropriate use of such drugs, increasing attention by the media and professional organizations, and the Centers for Disease Control and Prevention national campaign may have contributed to the observed trend toward declining antimicrobial use. Progress toward decreasing inappropriate use is being made in many states, although antimicrobial prescribing rates remain excessive for bronchitis, and use of broad-spectrum antimicrobial drugs is increasing nationally (22,27,33). State and local organizations should consider a balanced approach that includes limited statewide educational activities with increasing emphasis on local, provider-level interventions and policy development. These activities might include academic detailing by physician opinion leaders, feedback on antimicrobial prescribing performance (including Health Plan Employer Data and Information Set measures), and economic incentives for careful antimicrobial use. These strategies may have the greatest effect if implemented as quality improvement initiatives in collaboration with the leadership of health plans and clinic organizations. Ongoing assessment of prescribing trends and rates of antimicrobial drug resistance will be needed to measure the ultimate effect of these efforts.
